# Changes in the Clinicopathological Demographics of Vulvar Cancer in Japan: Increasing Oldest-Old, Stage Shifting, and Decreasing Cohort-Level Survival †

**DOI:** 10.3390/jcm8122081

**Published:** 2019-11-29

**Authors:** Shin Nishio, Koji Matsuo, Takeo Shibata, Satoshi Yamaguchi, Hiroyuki Kanao, Kazuhiro Takehara, Nobuhiro Kado, Akiko Tozawa, Hideki Tokunaga, Tatsuya Matsunaga, Hisamori Kato, Koji Horie, Akira Kikuchi, Takayuki Enomoto, Mikio Mikami

**Affiliations:** 1 Department of Obstetrics and Gynecology, Kurume University School of Medicine, Kurume, Fukuoka 830-0011, Japan; 2 Division of Gynecologic Oncology, Department of Obstetrics and Gynecology, University of Southern California, Los Angeles, CA 90033, USA; 3 Department of Health Management, Tokai University, Hiratsuka, Kanagawa 259-1292, Japan; tshibata@is.icc.u-tokai.ac.jp; 4 Department of Gynecologic Oncology, Hyogo Cancer Center, Akashi, Hyogo 673-0021, Japan; s-yama@hp.pref.hyogo.jp; 5 Department of Gynecologic Oncology, Cancer Institute Hospital of JFCR, Tokyo 135-0063, Japan; hiroyuki.kanao@jfcr.or.jp; 6 Department of Gynecologic Oncology, National Hospital Organization Shikoku Cancer Center, Matsuyama, Ehime 791-0245, Japan; katakehara@shikoku-cc.go.jp; 7 Department of Gynecologic Oncology, Shizuoka Cancer Center, Shizuoka 411-0934, Japan; n.kado@scchr.jp; 8 Department of Obstetrics and Gynecology, St. Marianna University School of Medicine, Kawasaki, Kanagawa 216-8511, Japan; a2ono@marianna-u.ac.jp; 9 Department of Obstetrics and Gynecology, Tohoku University School of Medicine, Sendai, Miyagi 980-8574, Japan; tokunagahideki@med.tohoku.ac.jp; 10 Department of Obstetrics and Gynecology, Yokohama City University Hospital, Yokohama, Kanagawa 236-0004, Japan; tyamatsu@yokohama-cu.ac.jp; 11 Department of Gynecology, Kanagawa Cancer Center, Yokohama, Kanagawa 241-8515, Japan; katohh@kcch.jp; 12 Department of Gynecology, Saitama Cancer Center, Saitama 362-0806, Japan; k-horie@cancer-c.pref.saitama.jp; 13 Department of Gynecology, Niigata Cancer Center Hospital, Niigata 951-8133, Japan; akirak@niigata-cc.jp; 14 Department of Obstetrics and Gynecology, Niigata University School of Medicine, Niigata 951-8510, Japan; enomoto@med.niigata-u.ac.jp; 15 Department of Obstetrics and Gynecology, Tokai University Hospital, Isehara, Kanagawa 259-1193, Japan; mmikami@is.icc.u-tokai.ac.jp

**Keywords:** vulvar cancer, trends, demographics, elderly, survival, Japan

## Abstract

Background: To examine trends in the clinicopathological characteristics of vulvar cancer in Japan. Methods: This is a nationwide retrospective study examining consecutive women with vulvar cancer between 2001 and 2010 in Japan (*n* = 1061). Temporal trends in demographics, tumor characteristics, and survival were assessed by cohort-level analysis. The National Cancer Institute’s Surveillance, Epidemiology, and End Result Program was used for external validation (*n* = 10,154). Results: The number of oldest-old women aged ≥80 years significantly increased (from 18.0% in 2001 to 30.6% in 2010; 70.5% relative increase) in the study period. A stage shift was observed, with stage I disease decreasing from 43.0% to 34.0% (21.0% relative decrease), and tumors with distant metastases increasing from 23.2% to 35.6% (53.3% relative increase, *p* < 0.05). The number of women who underwent surgical treatment decreased from 84.0% to 69.7% (17.0% relative decrease), whereas utilization of radiotherapy increased from 34.4% to 43.2% (25.7% relative increase) over time (*p* < 0.05). In the cohort-level analysis, the five-year survival rates significantly decreased from 2001 to 2010 (*p* < 0.05), specifically, 66.9% to 51.0% for progression-free survival (23.7% relative decrease), 79.5% to 67.9% for cause-specific survival (14.6% relative decrease), and 74.9% to 62.3% for overall survival (16.9% relative decrease). In the patient-level analysis, oldest-old women were less likely to undergo surgical treatment and were independently associated with decreased survival (*p* < 0.05). In the US cohort, the number of oldest-old women (25.2% to 27.8%) and the five-year cause-specific survival rate (81.8% to 79.9%) remained unchanged during the study period (*p* > 0.05). Conclusion: Demographics and outcomes of vulvar cancer in Japan significantly changed during the study period. An increasing oldest-old population and a stage shift to more metastatic disease resulted in a cohort-level decrease in survival rates.

## 1. Introduction

Vulvar cancer is a rare malignancy, accounting for 4% of all gynecological cancers; 65% of these cases occur in developed countries [1]. Historically, vulvar cancer was most commonly observed at a median age of 65–70 years [2]. The incidence of vulvar cancer in Japan is one-tenth to one-sixth of that in Western countries [2]. According to a systematic review [3], the pooled five-year overall survival (OS) rate is 64.9%, and patients with International Federation of Gynecology and Obstetrics (FIGO) stage I, II, III, and IV disease have five-year OS rates of 84.0%, 74.6%, 47.8%, and 9.4%, respectively. Vulvar cancer incidence rates have increased worldwide [4,5,6,7], and the incidence has increased with age [7].

The population in Japan has been significantly and steadily aging. In 2017, 27.7% of Japanese was 65 years old or older, and 13.8% was 75 years old or older [3]. In general, aging is a risk factor for gynecological cancer, and elderly patients often undergo suboptimal treatment, particularly for surgery, resulting in compromised survival. Because vulvar cancer is more common in elderly patients [8], the demographics of vulvar cancer in Japan are likely to change with time.

Vulvar cancer has been relatively understudied worldwide, including in Japan, with no large-scale clinicopathological investigations of vulvar cancer in the past three decades. Moreover, the published studies examined small samples, which interfered with data interpretation and utilization [9]. Therefore, large-scale studies are needed to understand the demographics and recent trends of vulvar cancer in Japan. Such findings may help establish optimal treatment strategies. The objective of this study was to examine populational trends in clinicopathological characteristics of vulvar cancer in Japan.

## 2. Materials and Methods

### 2.1. Study Sites

The study protocol was approved by the Ethics Committee of Kurume University (protocol number: 14034), which served as the host institution, and each participating Japanese Gynecologic Oncology Group (JGOG)-affiliated institution reviewed the protocol and approved the study. The study protocol was registered at the University Hospital Medical Information Network (UMIN) (protocol number: UMIN-000017080). The study concept and participation call were initially announced at all JGOG-designated institutions (181 sites), and 109 (60.2%) sites voluntary participated in the study. The study sites, however, covered all high-volume centers in Japan.

### 2.2. Data Collection

Upon completion of data collection by participating clinicians, the anonymous de-identified data sheets were transferred to the host institution. Data were then compiled into a master Excel data sheet by the research staff. The principal investigators reviewed the accuracy, consistency, and quality of the dataset, and when there was disagreement in a data element, the principal investigator and investigators at each participating institution discussed the context to achieve adjudication.

### 2.3. Patient Eligibility

Consecutive patients with invasive vulvar cancer from January 2001 to December 2010 were examined, and data curation of this retrospective observational study was conducted from August 2014 to March 2016. JGOG institutions are thought to see more patients with vulvar cancer, estimated at 5–10 patients per institution annually for 10 years. The inclusion criteria were a diagnosis of and treatment for invasive vulvar cancer, including patients whose initial therapy had a palliative intent and patients with primary vulvar cancer, including all histological types. Patients with malignant melanoma were excluded from the analysis. For eligible patients, information on patient demographics, tumor characteristics, surgical treatment, radiotherapy, chemotherapy, and survival outcomes was obtained from archived medical records.

### 2.4. Clinical Information

Patient demographics included age and year of diagnosis. Tumor characteristics included histological type (squamous, adenocarcinoma, or others), cancer stage, tumor size (2 cm increments), and surgical margin status (involved, <1, 1–2, or >2 cm). Surgical treatment included type of vulvar surgery (simple vulvectomy, partial radical vulvectomy, or radical vulvectomy), details of inguino-femoral lymphadenectomy (laterality, extent as resection versus dissection, unilateral/bilateral deep lymphadenectomy, number of harvested and tumor-involved nodes, and use of sentinel lymphadenectomy), and reconstructive surgery (partial skin graft, cutaneous flap, or myocutaneous flap). Information on perioperative complications was abstracted (wound dehiscence, edema, infectious lymphangitis, thrombosis, frequency, and grade of urinary tract infection).

Radiotherapy information included use in a neoadjuvant, adjuvant, definitive, or palliative setting. The details of radiotherapy were also abstracted, including radiation field, irradiation method (radical or palliative irradiation), linear accelerator (LINAC), electron beam, total dose, and treatment duration. Radiotherapy-related toxicity (both early and late) was also documented. Details on regimens and cycles of chemotherapy were obtained for patients who received concurrent chemoradiotherapy. Chemotherapy information included the setting (neoadjuvant, adjuvant, or salvage), regimen type, administered cycles, and treatment-related toxicity. Survival information included follow-up time, presence of recurrent/progressive disease, and vital status (death from vulvar cancer, death from other causes, or alive).

### 2.5. Study Definitions

Patients were grouped by age as non-older (<60 years), young-older (60–79 years), and oldest-old (≥80 years) per the World Health Organization/United Nations criteria (5). Cancer stage was reassigned based on the 2008 International Federation of Gynecology and Obstetrics (FIGO) criteria [6]. Progression-free survival (PFS) was defined as the time interval between vulvar cancer diagnosis and the first recurrence/progression of disease or death from vulvar cancer. Cause-specific survival (CSS) was defined as the time interval between vulvar cancer diagnosis and death from vulvar cancer. Overall survival (OS) was defined as the time interval between vulvar cancer diagnosis and death from any cause. Data on patients without survival events at the last follow-up visit were censored.

### 2.6. Study Aims

The primary objective of this study was to outline the time-specific descriptive statistics of the clinicopathological features of invasive vulvar cancer in Japan. The secondary objective was to assess the temporal trends of survival statistics and the prognostic factors of invasive vulvar cancer in the study population.

### 2.7. Statistical Considerations

Standard statistical methods were used to describe continuous variables as mean (±SD) or median Interquartile range (IQR) values based on normality, as assessed using the Kolmogorov–Smirnov test. Significance in differences among multiple groups (more than two) was assessed using one-way ANOVA or the Kruskal–Wallis *H* test, as appropriate. Discrete and categorical variables are displayed with numbers and percentages per group, and the statistical significance of differences was assessed with chi-squared tests.

For cohort-level analysis, the Joinpoint trend analysis software (version 4.4.0.0, National Cancer Institute, Bethesda, MD, USA) was used to examine temporal trends over time. Single calendar years were used as time increments, and percent frequencies with confidence intervals (CI) or observed values of 5-year survival rates with standard errors are reported. The 5-year survival time point was based on the median follow-up of the study cohort. Linear segmented regression was used to analyze temporal trends, and log-transformation was performed to determine annual percentage rate changes (APC) of the slope with 95% CI. Relative changes in outcomes were based on the modeled value in this study.

For the patient-level analysis, Cox proportional hazard regression models were fitted to assess the prognostic impact of the clinicopathological factors of interest on survival, and the magnitude of statistical significance was expressed using hazard ratio (HR) and 95% CI. Specifically, on the basis of the results of the cohort-level analysis, we examined the association of patient age and survival adjusted with a priori survival factors, histology, stage, tumor size, and treatment types. The year of diagnosis was also considered in the model. This parsimonious approach was due to the relatively small sample size of our study.

To examine if the cohort-level characteristics observed in the study population are unique to Japanese women, the National Cancer Institution’s Surveillance, Epidemiology, and End Result (SEER) Program was used for external validation (US cohort). Briefly, the SEER database is the largest population-based tumor registry in the United States, covering approximately 34.6% of the population in the latest version [10]. The cohort-level analysis was carried out to examine temporal trends in age, stage, and survival rates over time, as described earlier.

Various sensitivity analyses were performed to examine the robustness of the analysis, and trends and outcomes were assessed for stage I disease, surgery, and squamous histology. These subgroups were chosen on the basis of the rationale that vulvar cancer is more likely to exhibit squamous histology in early-stage disease, and surgical treatment remains the mainstay of treatment. In the statistical analysis, *p* < 0.05 was considered to indicate statistical significance (two-tailed hypothesis). The Statistical Package for Social Sciences (version 24.0, Armonk, NY, USA) was used for all analyses.

## 3. Results

### 3.1. Patient Characteristics

There were 1061 women who met the inclusion criteria (Table 1). The median number of vulvar cancer cases per site was 9.5 (IQR 5–14). The median age was 72 years, with 257 (24.2%) oldest-old patients. The tumors were more likely to be squamous (*n* = 768, 72.4%) and in stage I (*n* = 397, 37.4%). Metastatic tumors to inguino-femoral lymph nodes or distant organs were seen in 312 (29.4%) women. Most patients underwent surgical treatment (*n* = 800, 75.4%).

Regarding the surgical treatment, 273 (34.1%) patients underwent radical vulvectomy, 259 (32.4%) underwent some type of reconstructive surgery, and 494 (61.8%) underwent inguino-femoral lymphadenectomy. Tumors with close or involved surgical margins accounted for 436 (54.5%) cases. Perioperative complications were relatively infrequent (*n* = 100, 12.5%). Radiotherapy with any indication was performed in 410 (38.6%) women, with definitive (*n* = 194, 18.3%) and adjuvant (*n* = 168, 15.8%) radiotherapy being the two most common indications. Among women who received radiotherapy, concurrent chemotherapy was used in 103 patients (25.1%).

### 3.2. Demographics Trends

**Figure 1:**
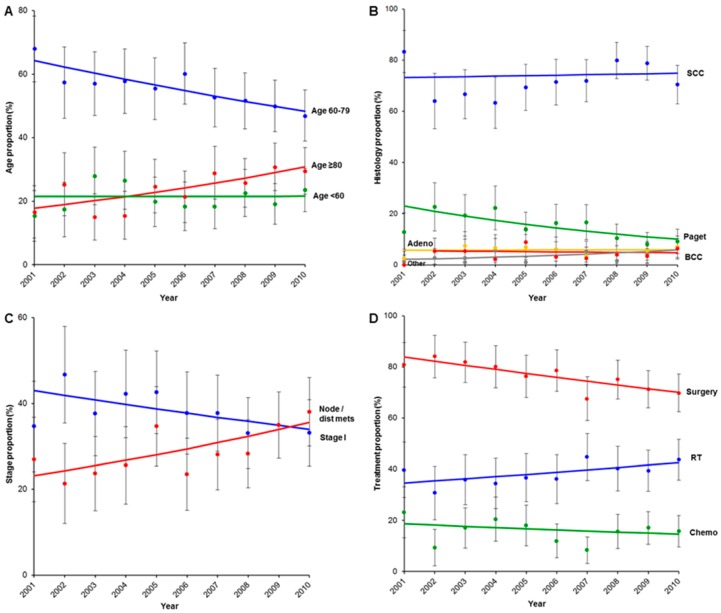
Temporal trends of vulvar cancer in Japan from 2001 to 2010. Temporal trends (**A**) age-specific (annual percentage rate changes (APC) for age 60–79, −3.0, 95% confidence interval (CI) −4.2 to −1.7, *p* < 0.001; and APC for ≥80, 6.1, 95% CI 1.6–10.8, *p* = 0.013), (**B**) histology type-specific, (**C**) stage-specific (APC for stage I disease, −2.6, 95% CI −4.9 to −0.2, *p* = 0.038; and APC for inguino-femoral nodal or distant metastasis, 4.9, 95% CI 1.2–8.7, *p* = 0.015), and (**D**) treatment type-specific (APC for surgery, −2.1, 95% CI −3.0 to −1.1, *p* < 0.001; and APC for radiotherapy (RT), 2.6, 95% CI 0.3–4.9, *p* = 0.032).

The number of oldest-old women significantly increased from 18.0% to 30.6% (70.5% relative increase), whereas the number of young-older women decreased from 64.0% to 48.8% (23.7% relative decrease) (for both, *p* < 0.05; Figure 1A). There was no change in histology type (Figure 1B). A stage shift was observed, and cases of stage I disease decreased from 43.0% to 34.0% (21.0% relative decrease), whereas cases of tumors with inguino-femoral nodal or distant metastasis increased from 23.2% to 35.6% (53.3% relative increase) (for both, *p* < 0.05; Figure 1C). The number of women who underwent surgical treatment decreased from 84.0% to 69.7% (17.0% relative decrease), whereas utilization of radiotherapy increased from 34.4% to 43.2% (25.7% relative increase) (*p* < 0.05; Figure 1D).

### 3.3. Cohort-Level Survival Trends

**Figure 2:**
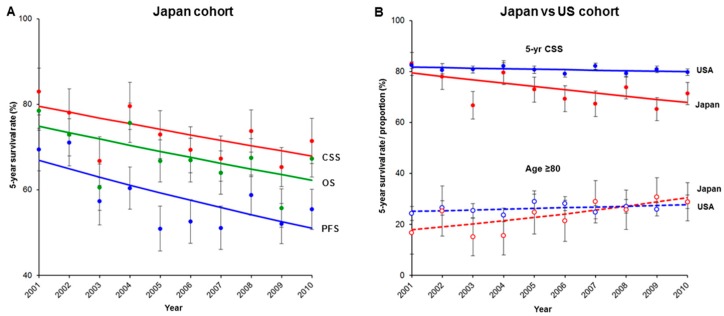
Cohort-level survival changes from 2001 to 2010. (**A**) Temporal trends for five-year progression-free survival (PFS), cause-specific survival (CSS), and overall survival (OS) rates from 2001 to 2010 for the Japan cohort, and (**B**) temporal trends for five-year CSS rates and proportion of women aged ≥80 years from 2001 to 2010 for the Japan and US cohorts.

The median follow-up of censored cases was 58.2 months (IQR, 23.6–86.2), with 423 cases of recurrence or progressive disease, 280 deaths from vulvar cancer, and 349 deaths from any cause during the follow-up. Among those who died, the median time to death was 14.5 months (IQR 7.2–37.8). Five-year survival rates significantly decreased (Figure 2A): 66.9% to 51.0% for PFS (23.7% relative decrease, APC −3.0, 95% CI −5.0 to −0.9, *p* = 0.012), 79.5% to 67.9% for CSS (14.6% relative decrease, APC −1.7, 95% CI −3.2 to −0.2, *p* = 0.029), and 74.9% to 62.3% for OS (16.9% relative decrease, APC −2.0, 95% CI −4.0 to −0.1, *p* = 0.045).

### 3.4. Patient-Level Survival Analysis

Patient demographics per age group are shown in Table 2. Oldest-old women were more likely to have squamous tumors (81.7% versus 66.2–70.6%) and close/involved surgical margins (75.3% versus 60.3–66.0%), but less likely to undergo surgical treatment (56.0% versus 77.7–91.9%), particularly radical vulvectomy (15.3% versus 37.4–38.9%), vulvar reconstruction (21.5% versus 34.4–36.0%), and inguino-femoral lymphadenectomy (43.8% versus 65.3–66.6%) than younger women (for all, *p* < 0.05). Oldest-old women were more likely to receive radiotherapy (46.1% versus 33.6–37.8%) and less likely to receive concurrent chemotherapy during radiotherapy (6.8% versus 28.3–45.2%) or systemic chemotherapy (4.7% versus 16.0–21.2%) than younger women (all, *p* < 0.05).

Similar trends were also observed for stage I disease only (Table 3). Despite the similar tumor stage and size across the age groups, women in the oldest-old group were less likely to receive optimal surgery-based treatments than women in the younger groups. On multivariable analysis (Table 4), oldest-old women had an approximately 60% higher risk of recurrence/progression of vulvar cancer (adjusted HR 1.586, 95% CI 1.139–2.209) and a roughly two-fold higher risk of all-cause mortality (adjusted HR 2.172, 95% CI 1.509–3.126) than non-older women (for all, *p* < 0.05). This association was also observed in subgroups with squamous tumors, stage I diseases, and surgery (Table 4). 

### 3.5. External Validation Cohort

In the US cohort (*n* = 10,154), the median age was 68 years (IQR 54–80), with 26.4% being oldest-old (*n* = 2683). The number of oldest-old women did not change from 2001 to 2010 (25.2% to 27.8%, APC 1.1, 95% CI −0.5 to 2.8, *p* = 0.160; Supplemental Figure S1A). The distribution of tumor stages was also unchanged during the study period (Supplemental Figure S1B).

The median follow-up of censored cases was 104 months (IQR 78–139), and during follow-up, there were 2046 deaths from vulvar cancer and 5433 deaths from any cause. Among those who died, the median time to death was 27 months (IQR, 10–62). The five-year CSS rate remained unchanged during the study period (81.8% to 79.9%, APC −0.3, 95% CI −0.6 to 1.0, *p* = 0.132; Supplemental Figure S1C). There was a slight decrease in the five-year OS rate from 62.7% to 58.5% (6.7% relative decrease, APC −0.8, 95% CI −1.4 to −0.1, *p* = 0.027).

When the two cohorts were compared (Figure 2B), the proportion of oldest-old in the Japan cohort was lower than that in the US cohort at the beginning of the study (18.0% versus 25.2%) but became higher at the end of the study period (30.6% versus 27.8%). In 2001, the five-year CSS rates were similar in the two cohorts (Japan versus US cohort: 79.5% versus 81.8%); in 2010, the five-year CSS rates in the Japan cohort and US cohort were 67.9% and 79.9%, respectively.

## 4. Discussion

Key findings of this study include that women with vulvar cancer in Japan have become significantly older and that tumors are more likely to metastasize outside the vulva. Accordingly, the survival of women with vulvar cancer in Japan has significantly decreased. Several points deserve further discussions.

First, women with vulvar cancer in our study population were significantly aging, thus reflecting the extremely aging population trend in Japan [11]. In Western countries, the number of women with vulvar cancer has increased, but the number of oldest-old has not changed [12,13,14]. This trend was also observed in the US validation cohort, making our finding unique. Generally, elderly patients are less likely to receive radical treatment due to their fragility or medical comorbidities [8], as we also observed in our study; in fact, oldest-old women with stage I disease were more likely to receive non-surgical treatment. Among those who underwent surgery, oldest-old women were less likely to receive radical surgery, presenting less adequate surgical resection margin. Collectively, our study clearly demonstrates the impact of aging in cancer management. As the global population ages, awareness of this phenomenon is necessary among care providers [15].

The second point is the stage shift to more advanced disease in Japan. No stage shift was particularly found in studies conducted in Western countries, including in our US validation cohort [12,13,14]. While we do not know the exact cause of this association, plausible explanations can be hypothesized. First, physiologically, aging is associated with immunosenescence, which may escalate tumor spread in elderly patients [16]. Second, patient compliance may affect the timing of seeking care for treatment. For instance, elderly women are less likely to have a breast cancer screening than younger women, resulting in delayed diagnosis [17]. In addition, delayed diagnosis is common for vulvar cancer and negatively impacts survival. Furthermore, proactive diagnostic intervention and biopsy may be necessary for a suspected vulvar mass and lesion. Society- and community-based efforts may help improve the awareness of early signs and symptoms of vulvar cancer for both care providers and patients.

The third point involves the decreased cohort-level survival, which may be associated with the increase in the oldest-old population and the stage shift to advanced disease; these two latter phenomena likely contributed the decreased five-year survival rates additively and synergistically. In Western studies [12,13,14], no changes in vulvar cancer mortality were observed in the US validation cohort. In our study, nearly 80% (280/349) of deaths were due to vulvar cancer; this is higher than what observed in the US cohort (37.7%; 2046/5433). This adds new information to the literature, in that Japanese women with vulvar cancer are more likely to die from vulvar cancer than from other causes; this is likely because it is commonly thought that vulvar cancer is a disease of the elderly and that older women are likely to die from other causes. Nationwide efforts may be needed to improve the survival of women with vulvar cancer in Japan.

Finally, the effects of hospital experience and surgical volume on vulvar cancer management merit discussion. In our study, the annual median number of vulvar cancer cases at each institution was 9.5 (less than one per year per institution). As the incidence of vulvar cancer in Japan was lower than that in Western countries [2], this may simply lead to lack of experience, particularly for surgery. The current study findings support that surgical performance in Japan seems inferior to European quality.

For instance, the number of surgically treated women who underwent incomplete tumor resection in our study, evidenced by the presence of tumors in the surgical margin, was significantly higher than that reported in a recent European study (22.4% versus 9.9%) [18], in which the number of patients per hospital was significantly greater than that in our study: 1618 cases per 29 institutions over 10 years (5.5 versus 0.95 cases per institution per year) [18], implying that surgical volume affects surgical performance in vulvar cancer.

As high surgical volume is clearly associated with improved survival in other gynecologic cancer types, centralization of hospitals treating vulvar cancer may be necessary in Japan [19,20,21,22]. Other considerations may include demographic changes, community-based education and campaigns regarding vulvar cancer signs/symptoms, a prompt referral system to treating center, and establishment of a credentialing system for oncology centers and surgeons.

The strengths of our study include the large sample size and an external US validation cohort that showed clear contrasts. To our knowledge, vulvar cancer reports in Japan were available only for local specific geographic areas, with no national surveys [9,11]. We believe that our study is the first study to demonstrate nationwide trends in vulvar cancer in Japan. The detailed treatment information collected for this study enriched the quality of the analysis.

Nonetheless, our study has several limitations. First, the study has all the limitations inherent in a retrospective study. A salient unmeasured bias that can impact the outcomes includes medical comorbidity, frailty, performance status, surgeon’s experience, patient compliance, and the decision-making process, all of which most likely affect treatment approach, surgical performance, and survival. Second, while this study population covers diverse areas of hospitals in Japan, not all hospitals in Japan participated, resulting in a potential selection bias. However, as described earlier, the sites that participated in the current study represent all the high-volume centers in Japan, which mitigates the possible selection bias. Third, due to the relatively long duration (10 years) of this study, there may have been changes in treatment methods, resulting in non-uniformity of treatment. A summary of these geographic disparities is shown in supplemental Table S1.

## 5. Conclusions

In summary, the demographics and outcomes of vulvar cancer in Japan significantly changed during the study period: an increasing oldest-old population and a stage shift to more metastatic disease resulted in a cohort-level decrease in survival rates. The optimization of treatment strategies and screening methods will be useful to decrease mortality due to vulvar cancer in Japan.

## Figures and Tables

**Table 1:** Patient demographics (*N* = 1061). SCC: squamous cell carcinoma, BCC: basal cell carcinoma, CCRT: concurrent chemoradiotherapy.

Characteristics	Number (%)
No.	*N* = 1061
Age	72 (IQR 62–79)
<60	222 (20.9%)
60–79	582 (54.9%)
≥80	257 (24.2%)
Year	
2001–2004	336 (31.7%)
2005–2007	313 (29.5%)
2008–2010	412 (38.8%)
Histology	
SCC	768 (72.4%)
Paget	152 (14.3%)
Adenocarcinoma	59 (5.6%)
BCC	45 (4.2%)
Others	37 (3.5%)
Stage	
IA	135 (12.7%)
IB	262 (24.7%)
II	249 (23.5%)
IIIA	118 (11.1%)
IIIB	104 (9.8%)
IIIC	25 (2.4%)
IIINOS	4 (0.4%)
IVA	68 (6.4%)
IVB	96 (9.0%)
Tumor size (cm)	3.5 (IQR 2.4–5.3)
≤2.0	198 (18.7%)
2.1–4.0	362 (34.1%)
4.1–6.0	179 (16.9%)
6.1–8.0	84 (7.9%)
8.1–10.0	52 (4.9%)
>10.0	23 (2.2%)
Unknown	163 (15.4%)
Surgical treatment	
No	261 (24.6%)
Yes	800 (75.4%)
Vulva surgery type *	
Simple vulvectomy	227 (28.4%)
Radical vulvectomy	273 (34.1%)
Partial vulvectomy	297 (37.1%)
Unknown	3 (0.4%)
Vulva reconstruction type *	
None	538 (67.3%)
Cutaneous flap	173 (21.6%)
Partial skin graft	55 (6.9%)
Myocutaneous flap	31 (3.9%)
Unknown	3 (0.4%)
Surgical margin status (cm) *	
>2	110 (13.8%)
1–2	113 (14.1%)
<1	257 (32.1%)
Involved	179 (22.4%)
Unknown	141 (17.6%)
Inguino-femoral lymphadenectomy *	
Not performed	301 (37.6%)
Performed	494 (61.8%)
Unknown	5 (0.6%)
Sentinel lymph node evaluation **	
Not performed	451 (91.3%)
Performed	41 (3.9%)
Unknown	3 (0.6%)
Lymph node metastasis ***	
No metastasis	489 (64.9%)
Single metastasis	98 (13.0%)
Multiple metastases	167 (22.1%)
Perioperative complication *	
None	694 (87.0%)
Yes	100 (12.5%)
Unknown	4 (0.5%)
Lymphatic complication **	
No	473 (95.7%)
Yes	20 (4.0%)
Unknown	1 (0.2%)
Vulvar site complication *	
No	718 (89.8%)
Yes	78 (9.8%)
Unknown	4 (0.5%)
Radiotherapy	
No	643 (60.6%)
Adjuvant	168 (15.8%)
Neoadjuvant	19 (1.8%)
Definitive	194 (18.3%)
Palliative	29 (2.7%)
Unknown	8 (0.7%)
CCRT ^§^	
No	306 (74.6%)
Yes	103 (25.1%)
Unknown	1 (0.2%)
Systemic chemotherapy	
No	818 (77.1%)
Yes	152 (14.3%)
Unknown	91 (8.6%)

Number (%) or median (IQR) is shown. * among surgery cases. ** among lymphadenectomy cases. *** among assessed cases including non-vulvar surgery cases. ^§^ Among radiotherapy cases. Abbreviations: IQR, interquartile range; SCC, squamous cell carcinoma; BCC, basal cell carcinoma; and CCRT, concurrent chemoradiotherapy.

**Table 2:** Age-specific clinico-pathological characteristics (*N* = 1061).

Characteristics	<60	60–79	≥80	*p*-Value
No.	*n* = 222	*n* = 582	*n* = 257	
Year				0.010
2001–2004	75 (33.8%)	201 (34.5%)	60 (23.3%)	
2005–2008	87 (39.2%)	239 (41.1%)	111 (43.2%)	
2009–2011	60 (27.0%)	142 (24.4%)	86 (33.5%)	
Histology				<0.001
SCC	147 (66.2%)	411 (70.6%)	210 (81.7%)	
Paget	18 (8.1%)	33 (5.7%)	8 (3.1%)	
Adenocarcinoma	9 (4.1%)	28 (4.8%)	8 (3.1%)	
BCC	20 (9.0%)	13 (2.2%)	4 (1.6%)	
Others	28 (12.6%)	97 (16.7%)	27 (10.5%)	
Stage				0.432
I	97 (43.7%)	212 (36.4%)	88 (34.2%)	
II	47 (21.2%)	134 (23.0%)	68 (26.5%)	
III	47 (21.2%)	143 (24.6%)	61 (23.7%)	
IV	31 (14.0%)	93 (16.0%)	40 (15.6%)	
Tumor size (cm)				0.703
≤4.0	125 (56.3%)	306 (52.6%)	129 (50.2%)	
>4.0	68 (30.6%)	184 (31.6%)	86 (33.5%)	
Unknown	29 (13.1%)	92 (15.8%)	42 (16.3%)	
Surgical treatment				<0.001
No	18 (8.1%)	130 (22.3%)	113 (44.0%)	
Yes	204 (91.9%)	452 (77.7%)	144 (56.0%)	
Vulva surgery type *				<0.001
Simple vulvectomy	50 (24.6%)	137 (30.4%)	40 (27.8%)	
Radical vulvectomy	76 (37.4%)	175 (38.9%)	22 (15.3%)	
Partial vulvectomy	77 (37.9%)	138 (30.7%)	82 (56.9%)	
Vulva reconstruction type *				0.007
No	130 (64.0%)	295 (65.6%)	113 (78.5%)	
Yes	73 (36.0%)	155 (34.4%)	31 (21.5%)	
Surgical margin status (cm) *				0.027
>2	35 (20.1%)	67 (18.2%)	8 (6.8%)	
1–2	34 (19.5%)	58 (15.8%)	21 (17.9%)	
<1	55 (31.6%)	148 (40.2%)	54 (46.2%)	
Involved	50 (28.7%)	95 (25.8%)	34 (29.1%)	
Inguino-femoral lymphadenectomy *				<0.001
Not performed	70 (34.7%)	150 (33.4%)	81 (56.3%)	
Performed	132 (65.3%)	299 (66.6%)	63 (43.8%)	
Sentinel lymph node evaluation **				0.498
Not performed	116 (89.2%)	277 (92.6%)	58 (92.1%)	
Performed	14 (10.8%)	22 (7.4%)	5 (7.9%)	
Lymph node metastasis ***				0.774
No metastasis	106 (63.9%)	279 (65.8%)	104 (63.4%)	
Single metastasis	19 (11.4%)	58 (13.7%)	21 (12.8%)	
Multiple metastases	41 (24.7%)	87 (20.5%)	39 (23.8%)	
Perioperative complication *				0.845
None	176 (87.1%)	392 (87.1%)	128 (88.9%)	
Yes	26 (12.9%)	58 (12.9%)	16 (11.1%)	
Lymphatic complication **				0.190
No	128 (97.7%)	283 (94.6%)	62 (98.4%)	
Yes	3 (2.3%)	16 (5.4%)	1 (1.6%)	
Vulvar site complication *				0.484
No	178 (88.1%)	408 (90.7%)	132 (91.7%)	
Yes	24 (11.9%)	42 (9.3%)	12 (8.3%)	
Radiotherapy				<0.001
No	146 (66.4%)	360 (62.2%)	137 (53.9%)	
Adjuvant	46 (20.9%)	101 (17.4%)	21 (8.3%)	
Neoadjuvant	13 (5.9%)	4 (0.7%)	2 (0.8%)	
Definitive	14 (6.4%)	101 (17.4%)	79 (31.1%)	
Palliative	1 (0.5%)	13 (2.2%)	15 (5.9%)	
CCRT ^§^				<0.001
No	40 (54.8%)	157 (71.7%)	109 (93.2%)	
Yes	33 (45.2%)	62 (28.3%)	8 (6.8%)	
Chemotherapy				<0.001
No	156 (70.3%)	440 (75.6%)	222 (86.4%)	
Yes	47 (21.2%)	93 (16.0%)	12 (4.7%)	
Unknown	19 (8.6%)	49 (8.4%)	23 (8.9%)	

Number (percentage per column) is shown. Chi-square test for *p*-value. Significant *p*-values are emboldened; * among surgery cases; ** among lymphadenectomy cases; *** among assessed cases including non-vulvar surgery cases; ^§^ among radiotherapy cases.

**Table 3:** Clinico-pathological characteristics of stage I disease (age-stratified).

Characteristics	<60	60–79	≥80	*p*-Value
No.	*n* = 97	*n* = 212	*n* = 88	
Year				0.083
2001–2004	30 (30.9%)	84 (39.6%)	21 (23.9%)	
2005–2008	39 (40.2%)	83 (39.2%)	42 (47.7%)	
2009–2011	28 (28.9%)	45 (21.2%)	25 (28.4%)	
Histology				0.022
SCC	57 (58.8%)	118 (55.7%)	64 (72.7%)	
Non-SCC	40 (41.2%)	94 (44.3%)	24 (27.3%)	
Stage				0.085
IA	39 (40.2%)	74 (34.9%)	22 (25.0%)	
IB	58 (59.8%)	138 (65.1%)	66 (75.0%)	
Tumor size (cm)				0.593
≤4.0	62 (77.5%)	141 (79.2%)	55 (73.3%)	
>4.0	18 (22.5%)	37 (20.8%)	20 (26.7%)	
Surgical treatment				<0.001
No	0	11 (5.2%)	22 (25.0%)	
Yes	97 (100%)	201 (94.8%)	66 (75.0%)	
Vulva surgery type *				0.003
Simple vulvectomy	30 (31.3%)	75 (37.5%)	21 (31.8%)	
Radical vulvectomy	18 (18.8%)	42 (21.0%)	1 (1.5%)	
Partial vulvectomy	48 (50.0%)	83 (41.5%)	44 (66.7%)	
Vulva reconstruction type *				0.016
No	72 (75.0%)	144 (72.0%)	59 (89.4%)	
Yes	24 (25.0%)	56 (28.0%)	7 (10.6%)	
Surgical margin status (cm) *				0.152
≥1	34 (41.0%)	49 (31.4%)	14 (25.9%)	
<1	49 (59.0%)	107 (68.6%)	40 (74.1%)	
Inguino-femoral lymphadenectomy (stage IB) *				0.003
Not performed	27 (47.4%)	60 (46.9%)	36 (75.0%)	
Performed	30 (52.6%)	68 (53.1%)	12 (25.0%)	
Sentinel lymph node evaluation (stage IB) **				0.468
Not performed	24 (80.0%)	60 (88.2%)	11 (91.7%)	
Performed	6 (20.0%)	8 (11.8%)	1 (8.3%)	
Perioperative complication *				0.819
None	89 (92.7%)	181 (90.5%)	60 (90.9%)	
Yes	7 (7.3%)	19 (9.5%)	6 (9.1%)	
Radiotherapy				<0.001
No	88 (90.7%)	189 (89.6%)	70 (80.5%)	
Adjuvant	9 (9.3%)	12 (5.7%)	2 (2.3%)	
Definitive	0	10 (4.7%)	14 (16.1%)	
Palliative	0	0	1 (1.1%)	
CCRT ^§^				0.407
No	8 (88.9%)	20 (90.9%)	17 (100%)	
Yes	1 (11.1%)	2 (9.1%)	0	
Chemotherapy				0.002
No	76 (78.4%)	181 (85.4%)	79 (89.8%)	
Yes	11 (11.3%)	6 (2.8%)	0	
Unknown	10 (10.3%)	25 (11.8%)	9 (10.2%)	

Number (percentage per column) is shown. Chi-square test for *p*-value. Significant *p*-values are emboldened; * among surgery cases; ** among lymphadenectomy cases; *** among assessed cases including non-vulvar surgery cases; ^§^ among radiotherapy cases.

**Table 4:** Multivariable models for age and survival outcomes.

		Whole Cohort		SCC		Stage I		Surgery	
Outcome	Age	Adjusted HR (95%CI)	*p*-Value	Adjusted HR (95%CI)	*p*-Value	Adjusted HR (95%CI)	*p*-Value	Adjusted HR (95%CI)	*p*-Value
PFS	<60	1		1		1		1	
	60–79	1.436 (1.100–1.874)	0.008	1.666 (1.194–2.323)	0.003	2.046 (1.188–3.523)	0.010	1.409 (1.054–1.884)	0.021
	≥80	1.586 (1.139–2.209)	0.006	1.705 (1.153–2.521)	0.007	2.321 (1.139–4.732)	0.020	2.096 (1.424–3.086)	<0.001
CSS	<60	1		1	0.091 *	1		1	
	60–79	1.347 (0.966–1.877)	0.079	1.440 (0.970–2.137)	0.070	2.089 (0.730–5.979)	0.170	1.433 (0.982–2.119)	0.062
	≥80	1.546 (1.033–2.313)	0.034	1.650 (1.046–2.601)	0.031	3.269 (0.934–11.438)	0.064	2.289 (1.340–3.909)	0.002
OS	<60	1		1		1		1	
	60–79	1.513 (1.108–2.068)	0.009	1.540 (1.063–2.230)	0.022	1.754 (0.812–3.787)	0.152	1.596 (1.117–2.280)	0.010
	≥80	2.172 (1.509–3.126)	<0.001	2.319 (1.536–3.500)	<0.001	2.923 (1.171–7.292)	0.022	3.230 (2.031–5.137)	<0.001

Cox proportional hazard regression models for multivariable analysis. Significant *p*-values are emboldened. The association of age and survival outcome was adjusted for stage (I, II, III, or IV), histology (SCC versus non-SCC), tumor size (≤4.0 versus >4.0 cm), surgery (yes versus no), radiotherapy (yes versus no), systemic chemotherapy (yes versus no), and year of diagnosis (continuous). HR: hazard ratio. * among surgery cases.

## Supplementary Materials

The following are available online at https://www.mdpi.com/2077-0383/8/12/2081/s1. Figure S1: Trends and outcomes in the US cohort, Table S1: Trends in incidence and survival of vulva cancer in Japan, Europe, and North America.
