# Supplementary material for: Changes in the Clinicopathological Demographics of Vulvar Cancer in Japan: Increasing Oldest-Old, Stage Shifting, and Decreasing Cohort-Level Survival
Source: J Clin Med. 2019 Nov 29;8(12):2081. doi: 10.3390/jcm8122081 (PMC6947365; doi:10.3390/jcm8122081)
Supplement: Supplementary file 1 [file jcm-08-02081-s001.pdf]

Supplemental Figure S1. Trends and outcomes in the US cohort.

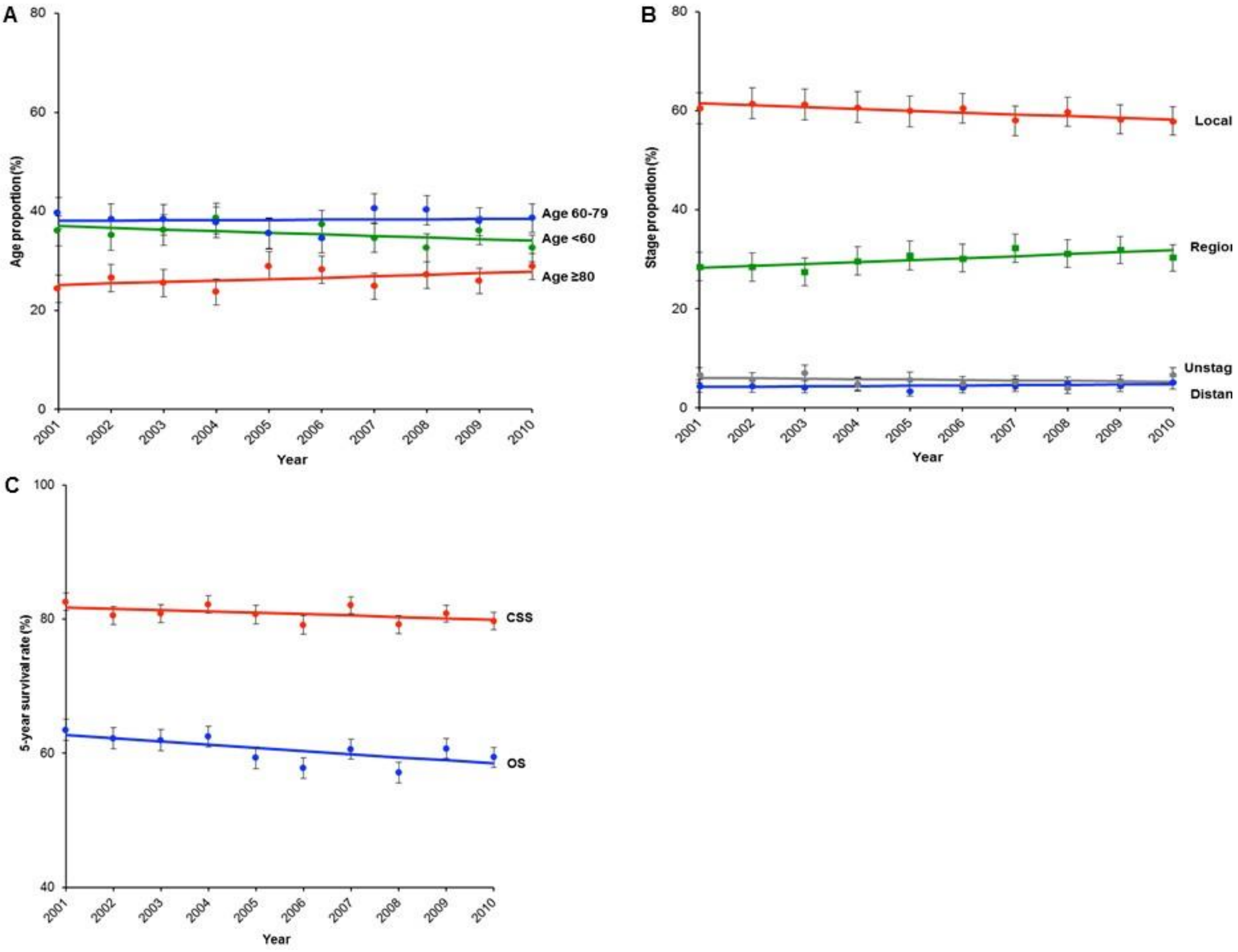

(A) Age trends, **(B)** stage trends, and **(C)** 5-year survival rate trends for the US cohort from 2001-2010. There was no statistically significant change for age and stage groups as well as CSS (all, $P>0.05$). The 5-year OS rates decreased from 62.7% to 58.5% (6.7% relative decrease, APC −0.8, 95%CI −1.4 to −0.1, $P=0.027$). Bold lines represent the modeled value, and circles represent the observed value with 95% CI. Abbreviations: CSS, cause-specific survival; OS, overall survival; APC, annual percent change; and CI, confidence interval.

Table S1. Trends in incidence and survival of vulva cancer in Japan, Europe, and North America

Characteristic	Japan	Europe	North America
Age	aging*	younger#	aging$
VIN	N/A	increasing‡	increasing†
Vulva cancer	↑advance stage*	↑early stage#	N/A
Survival	worsening*	no change#	no change$
Surgery	less performed*	N/A	N/A
Radiotherapy	more performed*	N/A	N/A
Chemotherapy	more performed*	N/A	N/A

N/A: not available, *:current research, #: Eur J Cancer 2013;49:3872-80,
$: Gynecol Oncol 2014;134:314-8, ‡: Eur J Gynecol Oncol 2001;22:393-402
†: Obstet Gynecol 2006; 107:1018-22
